# Heterogeneous Phenotypes of Primary Hyperparathyroidism in Romania: Characterization of a Large Cohort

**DOI:** 10.3390/jcm15103973

**Published:** 2026-05-21

**Authors:** Daniel Grigorie, Diana Felicia Coles, Alina Sucaliuc

**Affiliations:** 1 “C.I. Parhon” National Institute of Endocrinology, 011863 Bucharest, Romania; daniel.grigorie@umfcd.ro (D.G.); alina_sucaliuc@yahoo.com (A.S.); 2 Doctoral School of “Carol Davila”, University of Medicine and Pharmacy, 020021 Bucharest, Romania

**Keywords:** primary hyperparathyroidism, phenotypes, osteoporosis, fractures, TBS, nephrolithiasis, nephrocalcinosis, adenoma weight, diagnostic gap

## Abstract

**Background**: Primary hyperparathyroidism (PHPT) has undergone notable clinical changes over recent decades, with asymptomatic cases now prevailing in Western countries. In contrast, a broad spectrum of clinical manifestations remains common in Romania, an Eastern European country. This study aims to provide a representative descriptive analysis of clinical presentations and related complications observed in this setting. **Methods**: We performed a cross-sectional, single-center study of 413 consecutive PHPT cases diagnosed between 2000 and 2020 at a tertiary endocrinology center in Romania. Data included demographics, clinical features, biochemistry, bone turnover markers, 25OHD, BMD by DXA, TBS, fractures, renal involvement, and etiology. **Results**: Patients were predominantly female (88.6%), with a mean age of 60 ± 11.7 years and a mean BMI of 27.3 ± 5.7 kg/m^2^. Familial forms were identified in 4.4%. Mean serum calcium was 11.28 ± 1.09 mg/dL, mean PTH 248.31 ± 361.94 pg/mL, and mean 25OHD 17.95 ± 9.6 ng/mL. Symptomatic hypercalcemia was present in 23.2% and severe vitamin D deficiency in 21%. Fractures were present in 25.2% and osteitis fibrosa cystica in 1.7%. Mean T-scores (SD): LS –2.23, FN –1.85, 1/3 distal radius –1.96. Osteoporosis prevalence: LS 47%, FN 24.1%, 1/3 distal radius 38%. Mean TBS was 1.258 ± 0.115. Renal involvement included calcifications (56.7%), nephrolithiasis (53%), nephrocalcinosis (3.6%), hypercalciuria (31.7%), and reduced renal function (9.93%). Non-classical manifestations were mainly cardiovascular (58%) and osteoarticular (24.5%). Parathyroidectomy was performed in 217 patients (53%); histopathology showed adenoma (88.8%), carcinoma (5.2%), and hyperplasia (6%), with a mean adenoma weight of 2.86 ± 5.92 g. **Conclusions**: PHPT in Romania shows a heterogeneous phenotypic spectrum, reflecting variability in clinical presentation and suggesting an evolving epidemiological profile.

## 1. Introduction

Primary hyperparathyroidism (PHPT) ranks as the third most common endocrine disorder and is characterized by elevated serum calcium and inappropriately normal or high parathyroid hormone (PTH) levels due to autonomous PTH overproduction, leading to disturbances in mineral and bone metabolism, renal function, and neuromuscular physiology [1].

PHPT encompasses a broad biochemical and clinical spectrum. Three main clinical entities of PHPT are recognized, which coexist in varying proportions across different populations, influenced by the extent of biochemical screening and the prevalence of vitamin D deficiency [1,2]. Over recent decades, the clinical profile of PHPT has shifted from advanced, symptomatic disease, characterized by increased morbidity, skeletal deformities, and renal complications, to milder presentations identified through biochemical abnormalities, and alterations in bone density or quality. Overtly symptomatic forms of PHPT, marked by moderate (11.5–14 mg/dL) or severe (>14 mg/dL) hypercalcemia, proximal myopathy, and classical skeletal (osteoporosis with fractures, bone deformities, osteitis fibrosa cystica) and renal (calcifications, impaired renal function) involvement, are now uncommon in developed countries, accounting for approximately 20% of cases. In contrast, asymptomatic PHPT, predominant in Western countries (~80%), is typically associated with mild hypercalcemia (usually < 11.5 mg/dL), subtle or subclinical classical manifestations, and non-specific, non-classical symptoms, such as gastrointestinal, neuropsychiatric, neuromuscular, cardiovascular, and metabolic complaints. Regional variations persist: in China, the proportion of less severe forms has increased from 20% to 50%, whereas in India, asymptomatic cases still account for less than 5% [1,3,4]. The recognition of normocalcemic PHPT has also increased, following the wider use of biochemical screening, with reported prevalences ranging from 0.18% to 8.9% [5,6,7]. The phenotypic characterization of PHPT has further evolved to incorporate the presence or absence of target organ involvement, assessed after biochemical confirmation, as both mild hypercalcemic and normocalcemic forms may develop classical and non-classical complications affecting patients’ functional status and quality of life [1,2,3,4,8]. Most cases are sporadic, with approximately 85% due to parathyroid adenomas, while hyperplasia and carcinoma account for 10–15% and less than 1% of cases, respectively. Hereditary forms of PHPT are rare and may pose diagnostic challenges [1,2,3,8].

In Romania, there is still a significant gap in epidemiological data. However, small studies and clinical observations suggest a heterogeneous phenotypic spectrum of primary hyperparathyroidism, with a distribution that differs from current trends observed in Western countries [9]. This reflects the coexistence of asymptomatic forms (either hypercalcemic or normocalcemic, with or without target organ involvement) alongside persistent symptomatic presentations, including osteitis fibrosa cystica (OFC), a pattern more frequently reported in countries such as Pakistan or India. These characteristics may be related to the high prevalence of vitamin D deficiency (notably higher among Romanian PHPT patients compared to those with osteoporosis), limited access to modern parathyroid imaging techniques, and delayed diagnosis due to the absence of systematic biochemical screening [10,11,12,13].

In this study, we aimed to provide a comprehensive descriptive analysis of PHPT, focusing on the prevalence of its clinical and etiopathogenetic forms, as well as its associated classical (skeletal and renal) and non-classical manifestations.

## 2. Materials and Methods

### 2.1. Patients and Study Design

This is a single-center, cross-sectional observational study conducted at the “C.I. Parhon” National Institute of Endocrinology, Bucharest, Romania, a tertiary care center with nationwide referral coverage. The study was based on a retrospective analysis of a cohort of 413 consecutive cases diagnosed with primary hyperparathyroidism between 2000 and 2020. PHPT was defined by the presence of albumin-corrected serum calcium above 10 mg/dL in association with elevated or inappropriately normal parathyroid hormone (PTH) levels. Patients with secondary or tertiary hyperparathyroidism, familial hypocalciuric hypercalcemia (FHH), non–PTH-dependent hypercalcemia, and other metabolic bone diseases (except osteoporosis) were excluded.

### 2.2. Methods

The evaluation included demographic and anthropometric data (age at diagnosis, sex [F/M], body mass index [BMI]); clinical characteristics (years since menopause [YSM], diagnostic delay, symptoms of hypercalcemia, history of fractures, renal colic or surgery for urolithiasis, comorbidities); serum biochemistry (total calcium, phosphorus, magnesium, creatinine); hormonal and bone turnover marker [BTM] assessments (PTH; serum C-terminal telopeptide of type I collagen, osteocalcin, and procollagen type 1 N-terminal propeptide; vitamin D status assessed by 25(OH)D); urinary biochemistry (24-h urinary calcium and creatinine; urinary pH); bone parameters (areal bone mineral density [aBMD] assessed by dual-energy X-ray absorptiometry [DXA] and trabecular microarchitecture assessed by trabecular bone score [TBS]); fracture assessment (vertebral and nonvertebral fractures); renal involvement (calcifications, estimated glomerular filtration rate [eGFR], and cysts); and surgical and histopathological data.

Serum biochemical parameters were measured in fasting samples using standard automated laboratory methods (spectrophotometric techniques).

Urinary parameters were assessed using 24-h urine collections and spot urine samples. Urinary calcium and creatinine were measured using standard automated spectrophotometric methods, while urinary pH was determined using automated reflectometric strip methodology.

Serum 25-hydroxyvitamin D [25(OH)D], C-terminal telopeptide of type I collagen (CTX), osteocalcin (OC), procollagen type 1 N-terminal propeptide (P1NP), and intact parathyroid hormone (PTH) were measured using automated electrochemiluminescence immunoassays (Roche Diagnostics). Analyses were performed using successive generations of Roche analyzers over the study period.

Areal bone mineral density (aBMD) was measured at the lumbar spine L1–L4 (LS), femoral neck (FN), total hip (TH), and distal one-third radius (R) using DXA (iDXA, GE-Lunar, USA). In vivo precision, determined according to the standard procedures at our facility, was 1.2% at the lumbar spine, 1.5% at the femoral neck and 1.3% at the 1/3 distal radius. BMD results were interpreted according to the World Health Organization (WHO) criteria: normal BMD (T-score ≥ −1 SD), osteopenia (T-score between −1 and −2.5 SD), and osteoporosis (T-score ≤ −2.5 SD).

Site-matched lumbar spine trabecular bone score (TBS) was derived from DXA images using TBS iNsight software (v3.0.2.0, Medimaps Group, Geneva, Switzerland). For TBS interpretation, we used cut-off values recommended in the literature [14]: TBS > 1.350 indicates normal microarchitecture; TBS between 1.200 and 1.350 indicates partially degraded microarchitecture; TBS < 1.200 indicates degraded microarchitecture. TBS was calculated as the mean value of measurements from vertebrae L1–L4. Vertebrae excluded for BMD assessment were also excluded from TBS evaluation.

Vertebral fractures were primarily assessed using conventional lateral spine radiographs (T4–L4), obtained using a standardized technique. In patients with available imaging, vertebral fractures were diagnosed by visual assessment performed by the attending physician and independently confirmed by a radiologist using the Genant semiquantitative method. In a subset of patients without systematic imaging, vertebral fracture status was determined based on clinical history and available medical records. Non-vertebral fractures were assessed based on patient history (clinical interview). Osteitis fibrosa cystica was diagnosed based on radiographic evidence of subperiosteal bone resorption and bone cysts, with preserved cortical margins.

Renal involvement included nephrolithiasis, renal microlithiasis (defined as calculi ≤ 3 mm), nephrocalcinosis, and renal impairment (defined as eGFR < 60 mL/min/1.73 m^2^). These were assessed based on patient history, imaging studies (renal ultrasound or computed tomography, according to clinical indication and availability), and laboratory parameters, including serum creatinine, eGFR (calculated using the CKD-EPI formula), 24-h urinary calcium excretion, the calcium-to-creatinine clearance ratio (Ca/Cr Cl ratio), and urinary pH. Additional information included history of recurrent urinary tract infections (UTIs) and comprehensive urological or nephrological evaluation.

Parathyroid lesion localization was performed using cervical ultrasound, 99mTc-MIBI parathyroid scintigraphy, or SPECT/CT.

Genetic testing was performed in selected cases when a hereditary form was suspected. Familial forms were defined based on clinical, biochemical, imaging, and family history criteria.

Histopathological analysis was performed on surgical specimens after parathyroidectomy, including assessment of parathyroid tumor weight (in grams), histopathological type, and immunohistochemical (IHC) evaluation for parafibromin in selected cases (adenomas larger than 3.5 g or multiglandular lesions). Data were available only for patients with complete histopathological reports.

### 2.3. Statistical Analysis

Data were stored using Microsoft Excel and analyzed using JASP software (version 0.19.3.0; https://jasp-stats.org/ (accessed on 12 February 2026)). Data distribution was assessed for normality using the Shapiro–Wilk test or equivalent methods. Continuous variables were expressed as mean ± standard deviation (SD) or median, as appropriate according to data distribution. Categorical variables were expressed as numbers and percentages. Group comparisons were performed using Student’s *t*-test, ANOVA, or the Mann–Whitney test, as appropriate. A *p*-value < 0.05 was considered statistically significant. Informed consent for data collection was obtained from all patients.

## 3. Results

In the cohort of 413 patients with PHPT, 88.6% were female, with an F:M ratio of 7.7:1, a mean age of 59.96 ± 11.72 years, and a mean body mass index (BMI) of 27.35 ± 5.43 kg/m^2^. A high proportion of patients (65%) had a BMI ≥ 25 kg/m^2^. The diagnostic delay ranged from 0 to 16 years. At the time of diagnosis, the majority of female patients were postmenopausal with a mean menopause duration of 15 years.

Most patients had sporadic PHPT, while 18 patients (4.4%) had familial forms: MEN1 (11 patients), MEN2 (2 patients), Hyperparathyroidism-Jaw Tumor syndrome (HPT-JT) (3 patients), and isolated familial PHPT (2 patients).

The main biochemical parameters (mean ± standard deviation) are presented in Table 1, including serum calcium 11.28 ± 1.09 mg/dL, PTH 248.31 ± 361.94 pg/mL, 25OHD 17.95 ± 9.6 ng/mL, CTX 0.855 ± 1.04 ng/mL (as the cohort included patients treated with bisphosphonates), osteocalcin 50.82 ± 58.08 ng/mL, and P1NP 75.21 ± 30.31 ng/mL (N = 47). CTX values were significantly higher in patients not treated with bisphosphonates (n = 255; 0.946 ± 1.03 ng/mL) compared to patients treated with bisphosphonates (n = 148; 0.585 ± 0.491 ng/mL), with a significant difference (*p* < 0.001, Mann–Whitney test, r = 0.318). The prevalence of vitamin D deficiency (<20 ng/mL) was 60.5%, with severe deficiency (<10 ng/mL) in 21% of patients, while sufficient vitamin D status (≥30 ng/mL) was observed in only 9.9%.

Serum calcium levels were below 11.5 mg/dL in 67% of patients (n = 277), while 30.8% (n = 127) had values between 11.5 and 14 mg/dL, and only 2.2% (n = 9) had severe hypercalcemia (defined as values > 14 mg/dL).

Symptomatic hypercalcemia (polyuria, polydipsia, nausea, abdominal pain, anorexia, fatigue, bradyarrhythmias, and myopathy) was observed in 96 patients (23.2%), with a significantly higher mean serum calcium level (11.75 ± 1.38 mg/dL) compared with asymptomatic patients (11.11 ± 0.98 mg/dL; *p* < 0.001, r = 0.296, Mann–Whitney test).

Non-classical manifestations were distributed as follows: cardiovascular in 58% of patients (including hypertension and bradyarrhythmias); gastrointestinal in 20% (nausea, anorexia, constipation, epigastric pain, gastritis/ulcer, cholelithiasis, and pancreatitis); neuropsychiatric in 19.6% (fatigue, anxiety, mood or concentration disturbances, depression); neuromuscular in 3.6% (myopathy, 15 patients); osteoarticular in 24.9% (bone and joint pain, skeletal deformities); and disorders of glucose metabolism (type 2 diabetes mellitus and impaired glucose tolerance) in 12%. Overall, 47 patients (11.38%) had associated malignancies, most commonly thyroid cancer (53%; papillary: 11; medullary: 2) and breast cancer (12 patients).

To assess bone involvement in PHPT, we analyzed the presence of osteitis fibrosa cystica (OFC), fractures, osteoporosis (by DXA), and deterioration of trabecular bone microarchitecture (by TBS). OFC was identified in 7 patients (1.7%). Fractures were present in 25.2% of the entire cohort, predominantly nonvertebral (64.4%; 16.4% of the total cohort), including eight hip fractures. Vertebral fractures were identified in 7.2% of the entire cohort, while combined vertebral and non-vertebral fractures were observed in six patients (1.4%) (see Table 2).

After the first 153 patients, spine X-rays were systematically performed in the remaining 260 patients. Overall, 84 vertebral fractures (ranging from 1 to 6 per patient) were identified in the entire cohort. In the first 153 patients, imaging was performed selectively, and vertebral fractures were identified based on clinical history or radiographic evaluation in symptomatic cases.

Regarding bone mineral density (BMD), the mean T-scores placed most patients in the osteopenic range across all three evaluated sites. The highest prevalence of osteoporosis was recorded at the lumbar spine (N = 348, 47%), followed by the distal one-third radius (N = 21, 38%) and the femoral neck (N = 282, 24.1%) (see Table 3). Mean TBS was within the partially degraded range (1.258 ± 0.115); 32% had degraded microarchitecture (TBS ≤ 1.200), 51% had partially degraded microarchitecture (TBS > 1.200 and < 1.350), and 17% had normal TBS.

To assess renal involvement, we analyzed the presence of renal calcifications (including nephrolithiasis and nephrocalcinosis), hypercalciuria (24-h urinary calcium ≥ 0.3 g/24 h), impaired renal function (eGFR < 60 mL/min/1.73 m^2^), renal cysts and recurrent urinary tract infections (UTIs) (see Table 4). Renal calcifications were identified in 56.65% of patients. Nephrolithiasis was present in 53% of cases and included patients with microlithiasis (14.52% of the entire cohort), while symptomatic cases with a history of renal colic and/or urological interventions were identified in 17.19% of patients. Nephrocalcinosis was observed in 3.63% of patients.

The mean 24-h urinary calcium (available for 390 patients) was 0.25 ± 0.14 g/24 h. Hypercalciuria was present in 31.7% of patients. Unilateral or bilateral renal cysts were found in 6% of cases, while a history of recurrent UTIs was documented in 9.44% of patients. The prevalence of a urinary pH favorable to calcium phosphate and struvite stone formation (pH ≥ 6) was 22.3%, based on the 112 patients for whom data were available.

The mean serum creatinine value (N = 413) was 0.81 ± 0.25 mg/dL and reduced renal function (eGFR < 60 mL/min/1.73 m^2^) was observed in 9.93% of patients, predominantly corresponding to CKD stage 3; only one patient had an eGFR < 30 mL/min/1.73 m^2^.

The mean Ca/Cr Cl ratio (N = 106) was 0.02 ± 0.01. Although low ratios (<0.01) may suggest familial hypocalciuric hypercalcemia (FHH), all cases were ultimately classified as PHPT. Exclusion of FHH was challenging, particularly in the absence of genetic testing; however, these patients had longitudinal clinical and biochemical data supporting the diagnosis of PHPT. This finding reflects variable urinary calcium excretion, consistent with literature data describing the heterogeneity of this marker in PHPT.

Comparative biochemical data between patients with renal calcifications (nephrolithiasis: n = 219; nephrocalcinosis: n = 15 patients) and those without renal calcifications are summarized in Table 5.

Serum calcium levels were significantly higher in both the nephrolithiasis (Δ = 0.483 mg/dL, *p* < 0.001) and nephrocalcinosis groups (Δ = 1.671 mg/dL, *p* < 0.001) compared to patients without renal calcifications. Serum phosphate was significantly lower in the nephrolithiasis group (Δ = −0.218 mg/dL, *p* < 0.001), while the difference in the nephrocalcinosis group did not reach statistical significance (*p* = 0.084). 24-h urinary calcium was significantly higher in both the nephrolithiasis (Δ = 0.038 g/24 h, *p* = 0.013) and nephrocalcinosis groups (Δ = 0.104 g/24 h, *p* = 0.019) compared to patients without renal calcifications. Serum creatinine levels were significantly higher only in the nephrocalcinosis group (Δ = 0.280 mg/dL, *p* = 0.014). Mean eGFR was slightly lower in both the nephrolithiasis and nephrocalcinosis groups; however, these differences did not reach statistical significance (*p* > 0.05). The relatively small number of patients with nephrocalcinosis (n = 15) should be considered when interpreting these findings.

Regarding the normocalcemic form of PHPT (NC-PHPT), 12 patients (2.9%) (11 women and 1 man) met the diagnostic criteria after excluding other secondary causes of hyperparathyroidism. The mean age at diagnosis was 65.58 ± 7.31 years (range: 51–77 years), with a mean BMI of 27.79 ± 3.21 kg/m^2^, a mean serum calcium level of 10.19 ± 0.07 mg/dL, a mean PTH level of 89.37 ± 50.51 pg/mL, a mean LS T-score of −2.73 ± 0.93 SD, and a mean FN T-score of −1.71 ± 0.61 SD. Although NC-PHPT is often considered a milder form, organ involvement was observed in all cases. Notably, none of the patients had normal bone mineral density (BMD), and two patients presented with all three complications: low BMD, fractures, and nephrolithiasis. Non-classical manifestations were rare or absent. No neuromuscular (e.g., myopathy) or neuropsychiatric symptoms were observed. Glucose metabolism disorders (2 patients) and musculoskeletal symptoms (2 patients) appeared sporadically. Although cardiovascular manifestations such as hypertension and arrhythmias were reported in several patients (7 cases), they did not appear to be specific to this form of the disease.

To evaluate the etiology of PHPT, histopathological data were available for 115 patients with complete histopathological reports among those who underwent surgery (N = 217; 53%). Parathyroid adenoma was the predominant finding (88.78%, including 8 cases of double adenomas), followed by parathyroid carcinoma (5.2%, 6 patients), and parathyroid hyperplasia (6%, 7 patients). The mean adenoma weight, available for all 115 patients, was 2.86 ± 5.92 g (range: 0.05–48.6 g). The majority of parathyroid adenomas were small (≤2.5 g), accounting for 71.4% of cases. Nevertheless, large or giant adenomas or atypical parathyroid tumors (>3.5 g) were also identified in 16% of patients. In a subset of adenomas weighing more than 3.5 g, parafibromin staining was retained in all tested cases, supporting the diagnosis of adenoma rather than parathyroid carcinoma.

## 4. Discussion

To the best of our knowledge, this represents the largest single-center cohort of patients with primary hyperparathyroidism (PHPT) reported to date in Romania, providing insight into the phenotypic spectrum observed over recent decades and highlighting potential regional particularities. We characterized the cohort by evaluating both classical and non-classical manifestations of the disease, within the limits of the available data.

Biochemical screening demonstrated that PHPT is more prevalent than previously recognized, challenging its former characterization as a rare disease, a concept originally based on overt clinical manifestations such as acute severe hypercalcemia, nephrolithiasis/nephrocalcinosis, osteitis fibrosa cystica, and fractures [1,2].

In our cohort, a broad spectrum of clinical manifestations was observed. When considered in the context of previously published data from Western populations, where PHPT is predominantly asymptomatic, these findings suggest the coexistence of multiple phenotypic presentations. Consistent with the limited reports available from this region [10,11,12,13], the specific characteristics of PHPT in Romania remain incompletely defined. This may be partly explained by several factors, including the lack of population-based prevalence studies, the absence of long-term patient follow-up, limited access to advanced imaging techniques, and a shortage of experienced specialists in both diagnostic interpretation and parathyroid surgery. In addition, modern assays for calcium and PTH have become widely available only during the past two decades.

The latest international consensus on PHPT management [1] proposed clinical definitions for symptomatic and asymptomatic primary hyperparathyroidism. However, the classification based on the presence or absence of target organ involvement should be applied to asymptomatic PHPT only after biochemical confirmation, typically in screen-detected cases. The tertiary care setting may have contributed to the inclusion of more complex or atypical cases. Although our cohort largely consisted of clinically referred patients, access to medical evaluation in our setting is not strictly referral-based, as some patients are diagnosed through biochemical screening (serum calcium measurement) and may present directly for evaluation. Therefore, these definitions cannot be fully applied and potential selection bias should be acknowledged. Many cases were diagnosed during evaluation for osteoporosis or as part of the etiological investigation of nephrolithiasis, in accordance with current clinical practice. Consequently, for the purposes of this analysis, patients were classified as either symptomatic or asymptomatic based on the presence or absence of major clinical complications including symptomatic hypercalcemia, OFC, fractures, renal colic episodes, or chronic kidney disease.

Regarding normocalcemic primary hyperparathyroidism (NC-PHPT), we identified 12 patients (2.9%), whereas international studies have reported prevalence rates ranging from 0.18% [5,6,15] to as high as 8.9% in a Brazilian population [7].

We found that most PHPT cases in our cohort were sporadic, with only a small proportion representing familial forms, consistent with previous reports [3]. Familial forms included MEN1, MEN2, HPT-JT and isolated familial PHPT. Notably, one HPT-JT family carried a novel germline CDC73 mutation, as previously reported [16].

Non-classical complications in PHPT are often underestimated but may involve multiple systems, including neuropsychiatric, neuromuscular, cardiovascular, metabolic, and gastrointestinal systems. Although these manifestations may occur even in mild or normocalcemic forms, their severity varies considerably. In our cohort, non-classical and nonspecific manifestations were common, particularly cardiovascular and musculoskeletal symptoms. Their frequency was consistent with international reports; however, such manifestations are difficult to attribute exclusively to PHPT because they are highly prevalent in the general population [17,18,19,20,21,22,23,24,25,26,27,28,29,30,31].

The mean age at diagnosis of PHPT in our cohort was approximately 60 years, consistent with data reported in the literature, particularly from developed countries. The 60–80-year age group accounted for the largest proportion of PHPT cases (53%), likely reflecting population aging, increased life expectancy, and the presence of multiple comorbidities. PHPT remains an endocrine disorder with a marked female predominance; the female-to-male ratio in our cohort was 7.7:1, higher than the approximately 4:1 ratio commonly reported in the literature [1,3,32]. This discrepancy may reflect selection bias, as our clinic predominantly evaluated women with osteoporosis who were subsequently diagnosed with PHPT through standard diagnostic work-up. Epidemiological studies suggest that in developing Asian countries, PHPT tends to be diagnosed 15–20 years earlier than in Western Europe or North America. This age difference may be related to factors such as vitamin D deficiency and low dietary calcium intake, both associated with the development and growth of parathyroid adenomas [33,34,35,36].

Overweight and obesity are more frequent in patients with PHPT than in the general population [1,3], an observation also confirmed in our study, where 65% of patients were either overweight or obese. This finding may also reflect broader population trends, as obesity remains a major public health issue.

From a biochemical standpoint, the mean serum calcium and PTH levels in our cohort (calcium: 11.28 ± 1.09 mg/dL; PTH: 248.31 ± 361.94 pg/mL) fall between the averages reported in developed and developing countries. In the U.S. and Western Europe, reported mean values are 10.6 ± 0.6 mg/dL for calcium and 67.5 pg/mL for PTH (range: 47.9–94.9). In contrast, studies from Asia, Brazil, and South Africa have reported higher mean pooled values, with calcium levels of 11.9 ± 1.4 mg/dL and PTH levels of 668.6 ± 539 pg/mL [33,35,37]. The distribution of serum calcium levels in our cohort indicates that hypercalcemia was generally mild to moderate, with 67% of patients having calcium values < 11.5 mg/dL and only 2.2% presenting with severe hypercalcemia (>14 mg/dL). This pattern suggests a predominance of less severe disease forms, which may reflect evolving diagnostic practices or partial alignment with contemporary presentation patterns observed in more developed healthcare systems.

One of the severity criteria in PHPT is symptomatic hypercalcemia, which was more frequent in our cohort (23.2%) than rates reported in other studies (16%). This difference may be attributable to methodological, clinical, and contextual factors, including differences in screening practices and delayed diagnosis in our country [38]. These findings suggest that symptomatic forms of the disease remain clinically relevant in Romania.

Vitamin D deficiency is one of the key factors influencing the clinical presentation of PHPT. In our cohort, the prevalence of severe vitamin D deficiency (25OHD < 10 ng/mL) was 21%, within a national context characterized by endemic vitamin D deficiency before 2020. Patients with PHPT showed a higher rate of severe deficiency (35%) compared to patients with osteoporosis (22.2%). Previous national studies have reported an association between vitamin D deficiency and a more severe biochemical profile, although no clear association with a more aggressive clinical form of PHPT has been established [11,12].

Bone involvement in PHPT is now recognized as affecting not only bone mineral density (BMD) but also bone quality, as assessed by the trabecular bone score (TBS), involving both cortical and trabecular compartments. An increased risk of fractures at both vertebral and non-vertebral sites has been demonstrated in patients with PHPT [39,40]. Because BMD does not fully capture fracture risk, recent research has focused on bone microarchitecture; however, results regarding the clinical utility of TBS remain inconsistent. Some studies have reported an association between vertebral fractures and degraded bone microarchitecture (TBS < 1.200) [41]; however, our previous study did not confirm this association [39]. Consistent with those findings, more than 50% of patients in the current cohort were classified as having partially degraded microarchitecture.

The proportion of patients with low BMD (osteoporosis and osteopenia) across all measurement sites was approximately 80% in this study, consistent with the existing literature, although slightly higher than the 50–65% range reported in Western studies for individual skeletal sites and comparable to values of up to 85% reported in Eastern studies [17,42]. These findings suggest mixed bone involvement, with a high prevalence of osteoporosis at the lumbar spine and radius. However, results at the radius site should be interpreted with caution due to the small sample size. Overall, the data support impairment of both trabecular (lumbar spine) and cortical bone (radius, hip), which is characteristic of PHPT. Although skeletal involvement was notable in our cohort, classical severe manifestations such as osteitis fibrosa cystica and fractures were less frequent than osteoporosis. Fragility fractures were identified in 25.2% of patients, particularly among older individuals, supporting the role of age as an independent risk factor. Fracture distribution reflected predominant cortical bone involvement: non-vertebral fractures (16.4%), vertebral fractures (7.2%), and combined vertebral and non-vertebral fractures (1.4%). As spinal radiography was systematically performed in only 63% of patients, and vertebral fractures may remain clinically silent, their true prevalence was likely underestimated. Nevertheless, the observed rate remains clinically relevant when compared with findings from studies using systematic radiographic assessment [43]. The presence of OFC lesions (1.7% in our cohort, compared to < 5% in most reports but up to 22% in Indian cohorts) is generally considered an indicator of advanced disease [8,44].

Renal involvement, particularly renal calcifications identified in 56.65% of patients (53% with nephrolithiasis and 3.63% with nephrocalcinosis), may have been overestimated because of potential selection bias, particularly through the overdiagnosis of microlithiasis during ultrasound evaluation. The reported prevalence of symptomatic nephrolithiasis in patients with PHPT varies between 5% and 60%, depending on the diagnostic methods used (ultrasound versus CT), the biochemical severity of the disease, and cohort selection, whereas asymptomatic nephrolithiasis ranges between 11% and 35% [2,4,45,46,47]. When analyzed according to clinical presentation, symptomatic nephrolithiasis (renal colic) was present in only 17.19% of patients, while asymptomatic nephrolithiasis (including microlithiasis) was observed in 35.83%. The separate reporting of symptomatic and asymptomatic forms provides a more nuanced understanding of renal involvement and highlights the importance of systematic imaging together with clinical correlation in the evaluation of patients with PHPT.

The etiology of renal calcifications is multifactorial. Although hypercalciuria is present in about one-third of patients with PHPT and nephrolithiasis and is considered a major risk factor, it cannot be regarded as a standalone determinant. Other metabolic factors have increasingly gained attention for their role in shaping the lithogenic risk profile [4,48,49,50]. In line with most published data, our study found that 31.7% of patients with PHPT had hypercalciuria, defined as 24-h urinary calcium ≥ 0.3 g/24 h. The presence of nephrolithiasis and nephrocalcinosis was associated with a distinct biochemical profile. 24-h urinary calcium was significantly higher in both groups, supporting its role in the pathogenesis of renal calcifications. Serum calcium levels were significantly higher in patients with nephrolithiasis and even more elevated in those with nephrocalcinosis, consistent with greater biochemical disease activity. In addition, serum phosphate levels were significantly lower in nephrolithiasis and showed a similar downward trend in nephrocalcinosis, consistent with increased PTH-mediated renal phosphate excretion. The interpretation of impaired kidney function, estimated by serum creatinine and eGFR, remains complex, as it is unclear whether renal dysfunction is directly attributable to PHPT or to associated factors such as nephrocalcinosis, urinary tract infections, or previous urological interventions [50,51,52,53]. In our study, reduced renal function (eGFR < 60 mL/min/1.73 m^2^) was identified in 9.93% of patients with PHPT, with no cases among normocalcemic forms. This proportion was slightly lower than that reported in studies on mild or asymptomatic PHPT forms (12–17%), in which no significant differences in renal function were observed between patients with or without renal calcifications [51,54]. By contrast, symptomatic PHPT, particularly in patients with severe hypercalcemia (Ca > 14 mg/dL), has been associated with substantially higher rates of renal impairment, ranging from 26.2% in European studies to 30–56.5% in Indian cohorts [55,56,57,58]. Notably, in our cohort, mild renal impairment was observed only in patients with nephrocalcinosis: serum creatinine levels were significantly increased, whereas eGFR values were lower but did not reach statistical significance.

Regarding other renal associations, the reported prevalence of renal cysts in sporadic PHPT ranges from 3–10% and is not considered a typical manifestation [59,60,61]. Our finding (6%) falls within this range and likely reflects age-related changes or incidental imaging findings. Urinary tract infections (UTIs) are not direct manifestations of PHPT but may occur secondary to nephrolithiasis, particularly in the presence of urinary obstruction, or may themselves contribute to stone formation. Reported rates of recurrent UTIs in nephrolithiasis cohorts range from 8–15% [62]. The 9.44% prevalence observed in our study is consistent with these reports and may be related to the high prevalence of nephrolithiasis, which was identified in more than 50% of patients.

Histopathological data obtained from surgically treated patients (53% of the cohort) confirmed that parathyroid adenoma was the main cause of PHPT, being identified in 88.78% of cases, consistent with international reports [1,3,32,63]. The small proportion of parathyroid hyperplasia (only 6% of cases) may reflect selection bias, particularly the referral for surgery of patients without adequate preoperative localization of multiglandular disease. In contrast, the prevalence of parathyroid carcinoma (5.2%) was considerably higher than the < 1% typically reported in the literature [1,3,32]. This may be partly explained by the tertiary care setting and the inclusion of patients with hereditary syndromes. Notably, in one HPT-JT family, two of the three affected members were diagnosed with parathyroid carcinoma. Although based on a small number of cases, this observation is consistent with the increased malignancy risk reported in HPT-JT and may also have been influenced by the novel germline CDC73 mutation identified in this family [16].

Although the predominance (71.4%) of relatively small parathyroid adenomas (≤2.5 g) in our cohort is consistent with the modern PHPT phenotype described in Western countries, the persistent occurrence of large, giant, and occasionally atypical parathyroid tumors, accounting for 16% of cases and including lesions weighing up to 48.6 g, remains noteworthy given their reported rarity (1.5% of all parathyroid adenomas) [2]. These tumors, typically associated with more severe disease, were substantially more frequent than those reported in contemporary Western series (<6.5%). Bhan et al. [64] compared adenoma weight in two large cohorts from the USA and India (more than 400 patients in each group) and reported a mean adenoma weight of 1.26 ± 2.63 g in the USA cohort versus 4.53 ± 6.65 g in the Indian cohort (*p* < 0.001). Consequently, despite the predominance of smaller lesions, the mean adenoma weight in our cohort (2.86 ± 5.92 g) appeared intermediate between values reported in Western and Indian populations, being more than twofold higher than those described in USA cohorts and approaching those reported in Indian series. While some studies have suggested an inverse correlation between vitamin D levels and adenoma size, our previous studies did not confirm this association, although larger adenomas were associated with a more severe biochemical profile [12].

In this section, we contextualize our findings within the existing literature on primary hyperparathyroidism, highlighting both country-specific features and patterns described in other populations.

The clinical and biochemical spectrum of PHPT in our cohort ranged from asymptomatic forms, either normocalcemic or associated with mild hypercalcemia, to severe symptomatic disease characterized by marked hypercalcemia.

Our findings suggest that truly symptomatic PHPT accounted for up to 25% of cases and included symptomatic hypercalcemia, OFC, fractures, CKD, and symptomatic nephrolithiasis (renal colic). The presence of these complications highlights a disease profile combining features of both classical and more contemporary PHPT presentations.

By contrast, mild hypercalcemia, asymptomatic nephrolithiasis, and reduced bone mineral density without fractures were considered features of asymptomatic PHPT, accounting for up to 70% of cases.

To better contextualize these findings, we considered previously reported data, without performing a formal comparative analysis. Overall, our cohort demonstrated the coexistence of asymptomatic and symptomatic disease forms, reflecting a broad clinical and biochemical spectrum. From a biochemical perspective, our findings appear closer to patterns reported in some developing (Eastern) settings, whereas several clinical features resemble those described in Western populations. This observation is exploratory and warrants further investigation. This variability may be related to differences in diagnostic practices, the availability of biochemical screening, and access to specialized endocrine care and parathyroid surgery in a setting where delayed clinical presentation is still encountered.

This study has several limitations, including its single-center tertiary care design, retrospective cross-sectional nature, and potential selection bias, limiting causal inferences and broader epidemiological interpretation. Some evaluations were not systematically available for all patients. Nevertheless, the study provides a comprehensive characterization of PHPT in a large cohort recruited over an extended period, offering insights that may inform future research.

## 5. Conclusions

The clinical spectrum of PHPT in Romania remains heterogeneous, encompassing both asymptomatic and symptomatic forms. These findings highlight the variability in disease presentation and suggest an evolving epidemiological profile.

## Figures and Tables

**Table 1:** Main characteristics of patients with PHPT.

Characteristic/(Normal Range)	N	Median	Mean ± SD	Min–Max
Age, (yrs)	413	62	59.96 ± 11.72	15–83
BMI, kg/m^2^	413	26.70	27.35 ± 5.43	15–60.17
Postmenopausal yrs	319	15	14.98 ± 8.86	0–40
Serum calcium (8.4–10.2 mg/dL)	413	11	11.28 ± 1.09	10.1-18
Serum phosphorus (2.5–4.5 mg/dL)	411	2.7	2.72 ± 0.61	1.31–4.4
Serum magnesium (1.6–2.6 mg/dL)	114	2	1.99 ± 0.19	1.4–2.5
Serum PTH (16–62 pg/mL)	413	132.55	248.31 ± 361.94	47.86–2812
Serum 25OHD (19.8–58 ng/mL)	407	17	17.95 ± 9.6	1.47–90.24
Serum creatinine (0.5–1.2 mg/dL)	413	0.75	0.81 ± 0.25	0.19–3.02
24-h urinary calcium (0.07–0.3 g/24 h)	390	0.24	0.25 ± 0.14	0.01–1.37
eGFR (mL/min/1.73 m^2^)	413	91.66	87.77 ± 19.88	17.44–137.1
Ca/Cr Cl ratio	106	0.02	0.02 ± 0.01	0.001–0.07
CTX (0.33–0.782 ng/mL)	403	0.59	0.855 ± 1.04	0.06–11.52
Osteocalcin (15–46 ng/mL)	403	33.08	50.82 ± 58.08	5.03–609

**Table 2:** Bone Involvement in patients with PHPT.

Subcategory	Patients, n	Patients, %
Osteitis fibrosa cystica	7	1.7%
Total patients with fractures	104	25.2%
- Vertebral (V)	30	7.2%
- Non-vertebral (NV) *incl*. Hip	68	16.4%
- Mixed (V + NV)	6	1.4%

**Table 3:** BMD involvement in patients with PHPT.

DXA Site	Mean T-Score (SD)	N	Osteoporosis *	Osteopenia *	Normal BMD *
Lumbar spine (LS)	–2.23 ± 1.36	348	47%	35%	18%
Femoral neck (FN)	–1.85 ± 0.99	282	24.11%	59.21%	16.68%
Total hip (TH)	−1.77 ± 0.85	282	20.56%	61.36%	18.08%
1/3 distal radius (R)	–1.96 ± 1.3	21	38%	42.85%	19.15%

* classification according to WHO criteria.

**Table 4:** Renal involvement in patients with PHPT.

Subcategory	Patients, n	Patients, %
Renal calcifications	234	56.65%
Nephrolithiasis (total)	219	53%
- Microlithiasis	60	14.52%
- Renal colic	71	17.19%
Nephrocalcinosis	15	3.63%
Renal cysts	25	6%
Hypercalciuria (≥0.3 g/24 h)	131	31.7%
eGFR < 60 mL/min/1.73 m^2^	41	9.93%
eGFR 30–59 mL/min/1.73 m^2^ (CKD stage 3)	40	9.69%
Urinary tract infections (UTIs)	39	9.44%
Urinary pH > 6	25	22.3%

**Table 5:** Comparative biochemical data between groups of patients with/without renal calcifications.

Parameter	Lithiasis Δ Mean	Lithiasis *p*	Lithiasis r *	Nephrocalcinosis Δ Mean	Nephrocalcinosis *p*	Nephrocalcinosis r *
Serum Ca	0.483	<0.001	0.239	1.671	<0.001	0.546
Serum Ph	−0.218	<0.001	0.203	−0.275	0.084	0.263
24 h Ur Ca	0.038	0.013	0.146	0.104	0.019	0.358
Serum Cr	0.072	0.078	0.1	0.280	0.014	0.373
eGFR	−3.483	0.149	−0.082	−5.230	0.452	−0.114

* Mann–Whitney test, r = effect size.

## Data Availability

Data are available on reasonable request.

## Abbreviations

The following abbreviations are used in this manuscript:

**Acronym**	**Full term**
25(OH)D	25-Hydroxyvitamin D
BMD	Bone Mineral Density
BMI	Body Mass Index
CKD	Chronic Kidney Disease
CKD-EPI	Chronic Kidney Disease Epidemiology Collaboration equation
CTX	Cross-linked C-telopeptide of type I collagen
DXA	Dual-energy X-ray Absorptiometry
FHH	Familial Hypocalciuric Hypercalcemia
FN	Femoral Neck
GFR	Glomerular Filtration Rate
HPT-JT	Hyperparathyroidism–Jaw Tumor syndrome
IHC	Immunohistochemistry
LS	Lumbar Spine
MEN	Multiple Endocrine Neoplasia
MIBI	Methoxy-isobutyl-isonitrile (Technetium-99m sestamibi)
N	Number of patients with available data
P1NP	Procollagen Type 1 N-terminal Propeptide
PHPT	Primary Hyperparathyroidism
PTH	Parathyroid Hormone
SD	Standard Deviation
SPECT-CT	Single Photon Emission Computed Tomography—Computed Tomography
TBS	Trabecular Bone Score
TH	Total Hip
USA	United States of America
WHO	World Health Organization
